# Viral Co-Infections and Antiviral Immunity in Honey Bees

**DOI:** 10.3390/v15051217

**Published:** 2023-05-22

**Authors:** Alice Mélusine Durand, Anne Bonjour-Dalmon, Eric Dubois

**Affiliations:** 1 National Research Institute for Agriculture Food and Environement, INRAE, UR 406 Abeilles et Environnement, Site Agroparc, 84914 Avignon, France; anne.bonjour-dalmon@inrae.fr; 2 French Agency for Food, Environmental and Occupational Health Safety, ANSES, 06902 Sophia Antipolis, France

**Keywords:** virus, interaction, synergism, immune system, co-infection, honey bee, *Apis mellifera*, *Apis cerana*

## Abstract

Over the past few decades, honey bees have been facing an increasing number of stressors. Beyond individual stress factors, the synergies between them have been identified as a key factor in the observed increase in colony mortality. However, these interactions are numerous and complex and call for further research. Here, in line with our need for a systemic understanding of the threats that they pose to bee health, we review the interactions between honey bee viruses. As viruses are obligate parasites, the interactions between them not only depend on the viruses themselves but also on the immune responses of honey bees. Thus, we first summarise our current knowledge of the antiviral immunity of honey bees. We then review the interactions between specific pathogenic viruses and their interactions with their host. Finally, we draw hypotheses from the current literature and suggest directions for future research.

## 1. Introduction

The sustainability of our agriculture greatly relies on the pollination services provided by bees [1,2]. The decreasing population of wild bees and the increasing overwintering mortality of honey bees are caused by multiple interacting stressors [3]. Thus, it is vital to better understand bee physiology and behaviour as a whole. Fortunately, over the past decade, great research efforts have gone into the study of the interactions between categories of stressors, namely pesticides, urbanisation of natural spaces, intensive agriculture and monoculture, climate change, parasites, and pathogens [4,5,6,7,8,9,10,11,12,13,14], and honey bee immune dynamics when facing them [15]. However, honey bee diseases, and some viral diseases, in particular, remain understudied.

Around 72 viruses have been detected in honey bees [16]. Most are considered commensal, but some can be qualified as pathogens. Briefly, pathogenicity occurs when the interactions between a biological entity and its host lead to detrimental effects (virulence) in the host [17]. As defined by the damage-response framework [18], pathogenicity can either occur directly through the action of the pathogen or through the host immune response [19]. Long-term evolutionary promiscuity between an organism and its host can also lead to mutualistic interactions, as seen in gut microbiota [20], and in viruses [21]. However, no microbe is a pathogen or a symbiont by essence. Pathogenicity and symbiosis are emergent and dynamical properties driven by an ecological context [17,22,23,24]. Indeed, in many cases, viruses appear to be commensal (covert infection) until a variation in key biotic or abiotic factors turns the virus into a pathogen, mainly by dramatically increasing its replication and triggering clinical signs in its host [25,26,27,28]. These overt infections, defined as a disequilibrium in host-microbial interactions, are commonly called ‘diseases’.

A wide diversity of viruses can be detected in honey bees; here, our review focuses specifically on the viruses related to five major honey bee viral species triggering overt infections and known as critical for honey bee health: *Sacbrood virus* (SBV) can disrupt the development of honey bee larvae [29,30]; *Black queen cell virus* (BQCV) can cause early death in queen pupae [31,32]; the deformed wing viruses (DWV, including the most frequently reported variant genotypes DWV-A and DWV-B) can alter wing development during the pupal stage, leading to lifespan reduction in adults and their inability to fly [26,33]; Chronic bee paralysis virus (CBPV) can cause whole-body trembling, complete body melanisation and paralysis in adult bees [34,35]; and lastly, *Acute bee paralysis virus* (ABPV and the related viruses, *Israeli acute paralysis virus* (IAPV) and *Kashmir bee virus* (KBV) together considered as the AKI-complex) can lead to paralysis in artificial infections and abrupt death in natural conditions [36,37].

To study and understand diseases, considering honey bees as holobionts is of particular interest [38] because the internal ecology of an organism performs a crucial part in the microbes therein becoming pathogenic. Holobiont entities are defined as the whole network of interactions within the microbial community of a host and between the microbial community and the host itself [39,40]. The hologenome theory suggests that an organism’s fitness is intricately linked not only to its own genome but also to all hosted genomes, from the mutualistic microbiota to the more or less integrated pathosphere [41]. This approach requires understanding the complex cooperative and competitive interactions between all genomes taking part in the holobiont. However, when choosing between a reductionist approach and a holistic view of biological systems, researchers are bound to be torn between experimental controllability and accurate representations of complexity [42]. Although both approaches have their limitations, both are necessary for an integrated view of living processes [43]. Nevertheless, most experimental studies on honey bee viruses only considered single-virus infections, and most studies quantifying multiple viruses have only been descriptive. Thus, in this review, we strive to identify key interactions between selected threatening, disease-causing honey bee viruses and between these viruses and their host to foster further research on viral interactions towards an integrated view of honey bee health. For this purpose, we will first focus on the host response to viral infections as a whole before going in-depth into specific virus-virus interactions.

## 2. Honey Bee Antiviral Immunity

**Figure 1:**
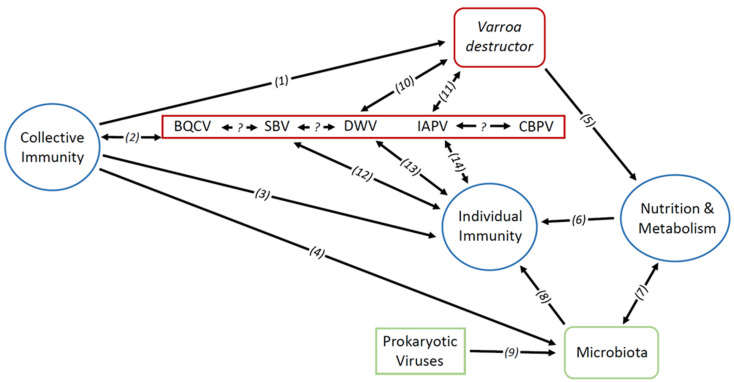
Schematic representation of the interactions between the main biological systems involved in honey bee virus dynamics. Unilateral arrows represent the influence of a system upon another system. Bidirectional arrows represent reciprocal influence between systems. Rectangles contain molecular organisms; rounded rectangles contain cellular organisms; circles contain physiological processes. Red shapes are considered pathogenic; green shapes are considered symbiotic or commensal; blue shapes are host-dependant systems. References: (1): [51,52]; (2): [51,52,53,54,55,56,57,58,59,60,61]; (3): [62,63,64,65,66,67]; (4): [68]; (5): [69,70,71]; (6): [72]; (7): [73,74,75]; (8): [76,77,78,79,80,81,82,83,84,85,86]; (9): [79]; (10): [7,87,88,89,90,91,92,93,94,95,96,97]; (11): [98]; (12): [99,100,101,102]; (13): [71,94,99,103,104,105,106,107,108,109]; (14): [110,111,112,113].

Viruses are obligate parasites; their replication thus depends on host metabolism, and they confront host defences. Most honey bee viruses and their hosts share a long evolutionary history that has led both sides to develop mechanisms to maintain their integrity and ensure their reproduction in a continuous arms race. Faced with viral infections, honey bees mobilise resources for physiological [44,45,46] and behavioural [47] immune responses. Therefore, viral interactions cannot be separated from host–parasite interactions and should be considered as a whole, intricate system [48]. Regarding physiological immune responses in insects, extensive work has been complete in drosophila [49] and mosquito [50] models, facilitating more recent inquiries into honey bee immunity. In this section, we first cover honey bee immune responses and the effectors involved in honey bee antiviral immunity. Figure 1 summarises the interactions between the main biological systems involved in honey bee virus dynamics.

### 2.1. Vitellogenin

Vitellogenin is a highly conserved egg yolk precursor protein [114]. Despite its functional origin being linked to queen fecundity [115], it performs a crucial role in the unfertilised honey bee workers as well [116]. As we demonstrate below, vitellogenin acts as an immune elicitor and regulates social task allocation and longevity in worker honey bees.

Vitellogenin and juvenile hormone act as mutual repressors. Together, they control age-related behaviour and task allocation [117]. Nurse bees display high vitellogenin stores and low juvenile hormone titres, whereas foragers display low vitellogenin stores and high juvenile hormone titres. In nurse bees, vitellogenin is metabolised in the hypopharyngeal glands for royal jelly production [116,118], used in turn for feeding worker and queen larvae. Later in life, increased expression of juvenile hormones leads to the degeneration of the fat body and the hypopharyngeal glands [119,120]. As these tissues serve as honey bees’ main vitellogenin production and storage sites [118,121], their degeneration leads to a depletion of vitellogenin stores [117]. This depletion in foragers leads to decreased longevity [122], increased gustatory responses [123], haemocyte apoptosis [124], and reduced resistance to oxidative stress [125]. This depletion has also been described following an artificial immune challenge, but heat challenges lead to increased vitellogenin expression [126]. This increased vitellogenin expression has been linked to decreased viral replication for DWV [127] and may explain the lower viral titres found in heat-challenged honey bees [128]. However, given the plurality of the physiological processes in which vitellogenin is involved, its expression cannot be considered a direct representation of honey bee immune activity. For instance, one study has shown that despite their low vitellogenin titres, foragers show increased anti-microbial peptide (AMP) production (see Section 2.3) compared with nurse bees [129], leading to a higher susceptibility to viral infection in nurses [130]. A similar pattern has been found in wintering bees, for which vitellogenin stores are preserved at the expense of a reduced immune response [131]. Vitellogenin is also involved in immune priming, as discussed in Section 2.6.

### 2.2. RNA Interference

The RNA interference (RNAi) pathway is the primary host defence against viruses in honey bees [46]. The first step of this immune pathway relies on Dicer proteins, a set of ribonucleases that recognise and cleave the intermediate double-stranded RNA (dsRNA) generated during the replication of RNA viruses. Dicer products are 21 to 23 nt RNA fragments called small interfering RNA (siRNA). The RNA-induced silencing complex (RISC) is able to detect these siRNAs and separate both RNA strands. The strand complementary to the targeted viral RNA is used as a probe by the RISC, specifically hybridising, then cleaving viral single-stranded RNA (ssRNA). Cleavage of viral ssRNA relies greatly on the activity of the Argonaute-2 protein (Ago-2), the catalytic component of RISC. In particular, the reduction in the *dicer* gene expression by gene knock-down leads to elevated replication of a model virus (*Sindbis virus* expressing the green fluorescent protein (GFP)) in honey bees [132].

Such RNAi mechanisms are known to be ancient. Indeed, not only has this mechanism been conserved across phyla [133,134], but the last eukaryotic common ancestor (LECA) likely already possessed this mechanism [135]. A recent study hypothesised that it actually appeared in the LECA as a dispensable regulatory system and not as a primary defence system [136]. The regulatory function of this system may still persist in contemporary insects. Although the core RNAi mechanism (i.e., RISC) relies on target-specific siRNAs [137,138], Dicer-2 activity may also trigger a non-specific dsRNA-mediated immune pathway [132]. Although the underlying mechanisms of this pathway are not known, in drosophila, Dicer-2 activates expression of the *vago* gene, which in turn produces a secreted protein activating the Jak-STAT pathway [139,140], a pathway involved in the regulation of other humoral immunity pathways through the expression of thioester-containing proteins (TEPs) [141] (see Section 2.3). This type of regulatory cascade may exist in honey bees, because DWV infection triggers *vago* expression [87] and the activation of the Jak-STAT pathway [103]. Alternatively, Dicer-2 products may also be involved in honey bee immune priming (see Section 2.6).

### 2.3. Humoral Immunity

Humoral immunity in insects comprises multiple pathways coordinating the production of AMPs [142]. Compared with RNAi, these pathways constitute a slower and less specific immune response [47]. The production of AMPs is mainly driven by the Toll and Immune-deficiency (Imd) pathways, both relying on an NF-κB transcription factor (Dorsal and Relish, respectively), whose continuous inhibition by IF-κB can be lifted to produce specific AMPs. The Imd pathway mainly controls the production of hymenoptaecins and apidaecins [143], and the Toll pathway mainly controls the production of defensins and abaecins [144], although these pathways crosstalk [145] and may both be involved in abaecin and defensin production [146,147]. The exact mechanisms of AMP effectors on viruses are currently not known, but it has been hypothesised that some AMPs can stabilise viral capsids, thus inhibiting viral RNA release and replication [148]. Feng et al. also suggested that the mechanisms of AMP action may cover the majority of the virus life cycle [149]. In contrast to the core RNAi mechanism, which is driven by specific virus targeting, the humoral immune pathways may be effective against all major honey bee viruses. Moreover, experiments on larvae and pupae suggest that each humoral pathway activation depends on the developmental stage in which the bee is infected and on the virus itself [99].

### 2.4. Melanisation

Viral infections can also trigger a melanisation response in honey bees. For instance, a melanisation response has been observed in DWV infections [99,104,150], although a study has found a negative correlation between DWV loads and melanisation response [105]. In this process, melanin acts as a physical barrier against pathogens. As such, honey bees naturally melanise their cuticle during development through phenoloxidase (PO) activity [151]. When uninfected larvae show little to no PO activity [152], this later increases in pupae [153] and adult honey bees [154] as they get older. In cases of infection, haemocytes are recruited to surround the recognised pathogens or infected cells [155]. Through PO activity, melanin is produced within haemocytes, which finally undergo apoptosis, thereby releasing melanin and encapsulating their target [156]. PO activity continues throughout the entire bee lifespan [157], maintaining melanisation of the cuticle. However, haemocyte counts significantly decrease when honey bees undergo their nurse-to-forager transition [157,158], although foragers’ encapsulation response remains unchanged [159]. For a more detailed review of cellular immune responses in honey bees, see [48].

### 2.5. Social Immunity

Within a honey bee colony, the high density of the honey bee population, coupled with the high genetic similarity between individuals, makes them particularly susceptible to parasites and pathogens [160], including viruses [161]. To further prevent pathogen outbreaks, social insects have developed alternative defence mechanisms known as social immunity [49,162]. Some studies have suggested that the development of social immunity in eusocial hymenopterans has led to decreased investment in individual immunity [144,163,164], although another study has not found evidence for a genetic trade-off between hygiene behaviour and individual immunity [165].

Most social immunity mechanisms are behavioural in nature. For example, honey bees can detect and dispose of sick or dead brood, whether it is infected by SBV [53], DWV [51,54], KBV [52], or infested by an ectoparasitic mite [51,52]. Interestingly, it has been suggested that the accumulation of DWV in the antennae [55] decreases this behaviour [51]. This hygiene behaviour towards brood is the target of selective breeding [166,167,168], and *Apis cerana* is known to exhibit quicker hygiene behaviour than *Apis mellifera* [56]. Honey bees can also detect virus-infected adult nestmates and exhibit either agonistic (biting, dragging out of the hive) or non-agonistic (allogrooming, antennation) behaviour towards infected individuals [57,58,59]. Additionally, infected, and immune-challenged honey bees reduce their trophallaxis behaviour towards nestmates [60], and queens may interact less with IAPV-infected workers [61].

As a more systemic mechanism, honey bees from different castes spatially distance themselves from each other when the colony is threatened by parasites, with foragers appearing less frequently on brood frames [169]. This type of distancing strategy can even lead infected individuals to banish themselves from the colony [170]. Honey bees are also known to increase in-hive temperatures following fungal infection by *Ascophaera apis* [171], although the effectiveness of this behaviour in preventing infection has been debated [172]. High temperatures can be detrimental to honey bee survival but can decrease DWV loads [128], although they do not seem to trigger individual immune responses [173]. However, it is currently unknown whether honey bees trigger a colony fever following viral infection.

In addition to the regulation of social interactions, social immunity includes the use of antiseptic substances as well. For instance, honey bees use Defensin-2 as a personal immune effector but secrete Defensin-1 in hive products, such as honey and royal jelly [174], and secrete more of it after being exposed to a pathogen [175]. Honey bees also coat the inside of the hive with propolis, an antiseptic substance made from resins [176,177], which they forage more actively following chalkbrood infestation [178]. The antiviral effects of propolis have been found against human viruses (reviewed in [179]); however, its effect on honey bee viruses is less clear. Some authors report no differences in DWV, BQCV, and IAPV loads between propolis-rich and propolis-poor colonies [176], but others indicate a higher increase in DWV loads in propolis-deprived colonies under varroa pressure [177]. Owing that propolis is made from various resins, these discrepancies may be due to substantial variations in propolis composition and require more investigation to identify candidate molecules with antiviral properties, whether they act directly or indirectly through microbiota enhancement [68] (see Section 2.7).

### 2.6. Immune Priming

Until recently, it was believed that insects rely solely on their innate immunity and lack acquired immunity mechanisms. While it is true that they mostly rely on innate immunity, evidence shows that many insects [180], including honey bees [62], are able to transfer pathogen fragments within and across generations, becoming agents for immune priming. Immune priming occurs horizontally when honey bee workers feed larvae with royal jelly [175]. To produce royal jelly, nurse bees mobilise vitellogenin, which acts as a transporter of immune primers, such as *E. coli* fragments [181] or viral dsRNAs [63], sending them to their hypopharyngeal glands, where vitellogenin is digested for jelly production [116]. The viral dsRNAs may originate from the RNAi pathway activity, given that Dicer activity produces dsRNAs (see Section 2.2). These immune primers are then transmitted to larvae when honey bee nurses feed them. In addition to this horizontal immune priming, the pathogen exposure history of queens also affects their offspring through a transgenerational immune-priming mechanism. Offspring of honey bee queens [64] or bumblebee queens [182] previously challenged with a bacterial infection show greater immune responses and survival when challenged with the same pathogen than offspring of unchallenged queens. The ‘suppressed in ovo viral infection’ (SOV) trait described by De Graaf et al. [65] may result from transgenerational immune priming against DWV and may prevent its vertical transmission. However, the effects of queens’ DWV infection history on transgenerational immune priming still seems limited [66] and depended on multiple factors, including viral infection routes [67].

### 2.7. Factors Altering Immune Function

Protein restrictions can impair immune responses [72] and promote viral replication [183,184,185]. Compared with colonies supplemented with polyfloral pollen, colonies supplemented with monofloral pollen show higher *Nosema ceranae* loads and lower tolerance to the microsporidia [186], and lower viral loads [187]. The importance of nutrition in honey bee health and immunity has rarely been considered in laboratory experiments. Bees in cage experiments often have access to sucrose solutions alone, but the inclusion of polyfloral pollen offers the best opportunity to depict natural interactions between the virobiome and its host.

In addition to hosting pathogens, honey bees host a great variety of non-pathogenic microbiota, which performs an important role in honey bee defences [76,77,78]. This microbial community also host their own virobiome, which would likely be involved in the holobiont homeostasis [79]. Although honey bee microbial community does not affect DWV loads after oral inoculation, it contributes to the bee tolerance to DWV infections [80]. Higher diversity in honey bee microbiome has also been linked to resistance to SBV in Korea [81], with some bacterial species identified as specific to SBV-resistant bees [82]. Finally, exposure to antibiotics was found to negatively alter honey bee gut microbiota [78,83], leading to increased susceptibility to IAPV infection [84]. These contributions of gut microbiota to host tolerance and resistance have been described as part of a co-immunity mechanism [85,86]. These mechanisms merit further study: a recent study reported suppression of a DWV infection and a decrease in mite survival after feeding honey bees with a modified gut bacterium, thereby stimulating the honey bee RNAi pathway [188]. Additionally, the composition of microbial communities of many bee species relies on their nutrition [73,74,75], confirming the key role of nutrition in bee health.

*Varroa destructor* infestation may also lead to an alteration of the honey bees’ immune response [104]. However, due to the complex link between varroa mite infestation and DWV infection (see Section 3.1), determining immune alterations specifically caused by the mite has been challenging [189]. For this reason, the underlying mechanisms of varroa-induced immunosuppression are still under debate. Varroa mites feed primarily on bee haemolymph and fat body tissue [69], which is the main vitellogenin production and storage site [118]. Multiple studies endeavouring to disentangle the effects of varroa and DWV on the immune response have not found evidence for an active varroa-mediated immunosuppressive mechanism [70,143,190]. Therefore, the observed alteration of the immune response following infestation may be more likely due to varroa feeding on the honey bee haemolymph and fat body, potentially leading to the depletion of vitellogenin stores [71] and detrimental effects on metabolism [70], which in turn have negative effects on immune responses (see Section 2.1).

Finally, honey bee genetics may also perform a key role in resistance to viral infections. For example, bees expressing the SOV genetic trait have proven to be more resilient to DWV infections [65]. Additionally, honey bee pupae from different patrilines within the same colony seem to show different patterns of DWV infection [191], which may indicate either a differential infection state between drones or a difference in genetic traits related to virus resistance.

## 3. Viral Interactions

**Figure 2:**
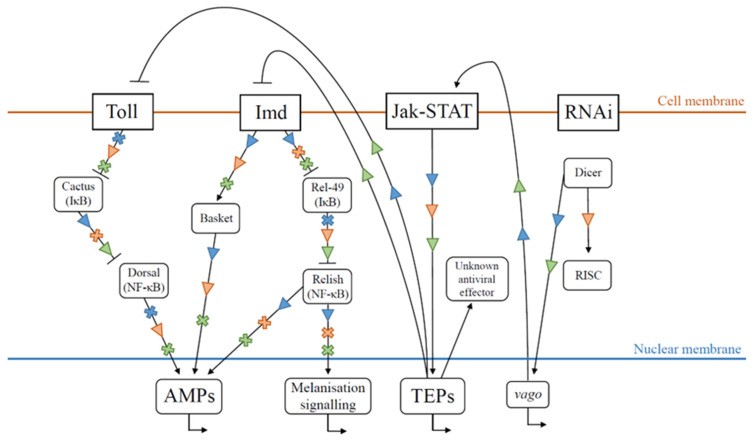
Simplified schematic representation of the hypothetical honey bee immune responses following single infections by either *Deformed wing virus* (DWV, blue), *Sacbrood virus* (SBV, orange), or *Israeli acute paralysis virus* and *Acute bee paralysis virus* (IAPV and ABPV, green). Black lines represent putative interactions, with black arrows indicating putative activation and blunt ends putative inhibition. Coloured arrowheads represent segment activation following viral infection, and coloured crosses activate segment inhibition following viral infection. References related to IAPV: Putative evasion of RNA-induced silencing complex (RISC) activity [110], Jak-STAT pathway activation [110], inhibition of Toll and Immune deficiency (Imd) pathways [110], Dicer activity [111], and melanisation activity [113]. References related to DWV: Jak-STAT pathway activation [103], Toll pathway inhibition [71,99], Imd pathway activation [103,107,108], melanisation activity [104], *vago* expression [87], putative evasion of RISC activity [94], and Dicer activity [111]. References related to SBV: Jak-STAT pathway activation [101], Toll and Imd pathway activation [99], melanisation pathway inhibition [99], and RNAi pathway activation [102]. AMP: Anti-microbial peptide; TEP: Thioester-containing protein.

In the previous section, we reviewed the mechanisms by which honey bees maintain their microbiome in a non-pathogenic state. In the following section, we will try to identify potential interactions between the major viruses threatening honey bee health. These interactions can be either direct or indirect [192]. For instance, direct interactions between virions can lead to genomic recombination [88,193]. Another type of direct interaction is superinfection exclusion (SIE), whereby an already established infection by one virus prevents subsequent infection by another virus [194,195]. Viruses can interact indirectly through their effects on host physiology, behaviour, or immune responses [196,197]. A graphical summary of honey bee individual immune pathways and their putative activation and inhibition by DWV, SBV, and AKI-complex viruses can be found in Figure 2.

### 3.1. DWV-A and DWV-B

DWV is a major threat to honey bee colony health and has been implicated in colony collapse disorder [198,199]. Although other master variants have been described (DWV-C [200], DWV-D [201]), their, respectively rare detection and disappearance incited research efforts to focus on the two most commonly detected master variants: DWV-A and DWV-B. The evolution and virulence of DWV appear to be closely related to the biology of the ectoparasite mite *V. destructor*. The varroa mite has been described as a biological vector of DWV [89,90], altering the viral process by providing a new route of infection. In previously spared countries, the introduction of the mite first led to an evolutionary bottleneck for DWV [91], followed by an increase in viral genomic diversity fostered by the mite [92,93,94,136]. As a result, a variant of DWV, originally described as varroa destructor virus 1 (VDV-1) [95] and later called DWV-B, has rapidly supplanted other variants in some parts of the world [39]. This variant can replicate within the mite [7,96], can replicate faster in honey bee pupae [202], and is thought to be more virulent than DWV-A in adult bees [203,204,205], although [206] reports similar virulence for both variants in pupae. Given the recent evolution of the virus and the threat, it represents for honey bees, understanding how new variants appear, their virulence and how they interact is critical for anticipating its future evolution and managing it effectively. Recombination events between DWV variants are indeed common [92] and occur at preferential recombination hotspots [88] in the genome. The varroa-guided selection seems to favour recombinants carrying non-structural genomic information from DWV-A and capsid genomic regions of DWV-B [88,97]. It has been suggested that this DWV-B genomic region has been favoured over its DWV-A counterpart because it may enhance transmission between varroa mites and honey bees.

Results regarding interactions between DWV variants and recombinants are less clear. Some studies have described an SIE of DWV-A by pre-infecting DWV-B [194], identifying genetic relatedness as a prominent factor in this interaction [195]. Recombinants may thus be an essential part of SIE between variants. However, in several studies, co-inoculation of multiple variants and recombinants did not alter either variant’s reproductive success [94,195,206]. Conversely, some studies suggest a potential helper effect between co-infecting variants [207,208], describing a higher DWV-B prevalence when co-infecting DWV-A is present. As discussed in [92,209], the timing of transmission, the viral background of infected bees and infection routes are critical in shaping the evolution of this virus.

### 3.2. DWV and SBV

As the importance of DWV in colony collapse disorder has become clearer, an increasing number of studies on this virus have revealed the potential that DWV has to interact with other viruses. In particular, interactions between DWV and SBV have been suggested. A study focusing on the transcriptomic profile of eggs laid by different queens [210] suggested that a synergistic effect between DWV and SBV explains the observed higher differential expression of genes between uninfected and co-infected eggs than in eggs infected by only one virus. Another study focusing on the outcomes of DWV inoculations [211] found outstanding SBV replication rates in pupae inoculated with some—but not all—DWV strains regardless of the inoculated variant (DWV-A or DWV-B), suggesting a strain-dependent helper effect favouring SBV replication and early pupae death. Similarly, a study on the change in the composition of DWV strains over the course of multiple serial inoculations in pupae [9] reported an increase in basal SBV and BQCV loads following injection of DWV, a trend that continued in subsequent serial injections to the point that SBV and BQCV outcompeted DWV, preventing DWV establishment. Finally, one study [99] directly compared the immune responses of bees inoculated with either SBV or DWV at either the larval or pupal stage and showed differential patterns of immune activation and inhibition that would suggest potential interactions between the two viruses. This latter study is of particular interest because, as we will show, knowledge of interactions between these viruses and their host may help determine the mechanisms behind DWV and SBV interactions.

DWV infections seem to inhibit the production of hymenoptaecin at larval stages [99] while triggering its activation in later stages through the Imd pathway [103,106,107]. Conversely, DWV does not interact with the Toll pathway in larvae [99], although in later stages, high DWV loads inhibit the production of defensins [99,104,105,106,107,108] and abaecin [104]. This inhibition is thought to be a consequence of the up-regulation of *cactus*, producing an inhibitor of Toll’s NF-κB effector, following a Domeless-dependent Jak-STAT pathway activation [103]. Varroa infestation triggers the Toll pathway in bees, but the presence of both varroa and DWV does not trigger this pathway, suggesting that DWV infection may actively inhibit the Toll pathway [71]. Given that Defensin-1 is used as a general social immunity effector secreted in hive products, its inhibition by DWV may facilitate replication in virtually all honey bee pathogens in the colony. Additionally, it has been suggested that some DWV strains may evade the RISC-mediated RNAi pathway [94]. However, a simultaneous increase in DWV loads and *dicer* and *ago-2* expression have been described in varroa-challenged bees [109]. As honey bees respond to SBV infections with the production of AMPs [99,100], inhibition of some humoral pathways induced by co-infecting DWV may have a helper effect on SBV replication. Conversely, SBV down-regulates the melanisation pathway at every stage of honey bee development [99]. Given that DWV infections trigger the melanisation process [104], this inhibition may lead to elevated DWV loads and virulence. However, because DWV vertical transmission relies on the survival of infected pupae, SBV down-regulation of the melanisation process may still be detrimental to DWV transmission. A summary of the various immune pathway activations and inhibitions caused by these two viruses can be found in Figure 2.

Here, it is important not to confuse synergistic with mutualistic interactions. A unilateral helper effect can, in the end, see the helped virus outcompete the helper virus or the development of mechanisms that prevent further replication of the latter. Evidence points to this type of situation in DWV-SBV interactions. DWV may have a helper effect on SBV replication, possibly through the inhibition of the Toll pathway. Although SBV replication may, in turn, help DWV replication through the inhibition of the melanisation pathway, the consequent increase in virulence may be detrimental to DWV transmission. Moreover, potential synergies between SBV and BQCV may make SBV a better competitor than DWV in the long run, as seen in [9].

### 3.3. SBV and BQCV

As we saw in the previous section, SBV and BQCV loads can increase concomitantly following DWV infections [9]. Evidence of co-prevalence between the two viruses has also been found [212]. A pioneering study [213] also found co-prevalence of these viruses, and a decreased ABPV prevalence when SBV and BQCV are both present, despite the outstanding competitive success of AKI-complex viruses [214]. Despite a clear lack of studies focusing on SBV-BQCV interactions, their shared infection patterns and correlated prevalence incite further research in this direction.

### 3.4. Viruses from the AKI Complex and Other Viruses

It has been strongly suggested that IAPV can produce a viral suppressor of RNAi (VSR) and thus evade RNAi immunity [110] (see Section 2.2). Indeed, a genomic sequence has been found to be conserved between IAPV and cricket paralysis virus (CrPV, also related to the *Dicistrovirus* genus), in which this sequence has been identified as coding for a VSR. Another study [111], upon finding 22 nt IAPV fragments (siRNA) following IAPV infection, suggests that this virus does not evade Dicer activity. Given that the action of the CrPV VSR focuses on the inhibition of RISC binding [215], finding *dicer* products following infection is still expected in IAPV infections. More surprising is the finding of an up-regulation of *ago-2* following IAPV infection [112], although this study did not report up-regulation of other RISC elements. IAPV also triggers the Jak-STAT pathway but not the Toll and Imd pathways [110]. Similarly, no AMP production could be detected after ABPV infection [113]. Co-infections between ABPV and *E. coli* trigger AMP production, but at a lower level compared with *E. coli* infections alone, suggesting that AKI infections actively inhibit the Toll and Imd pathways [113]. The immune pathway activations and inhibitions caused by AKI infections can be found in Figure 2.

No study has yet investigated the potential interactions between AKI-complex viruses and CBPV, but many clues suggest they exist. First, although they are not part of the same viral family, they both induce comparable symptoms when inoculated in adult bees [36]. Second, both viruses invade neural cells [216], even though IAPV seems to preferentially invade peripheral nerve cells [110], whereas CBPV is mostly found in central brain areas [217]. To our knowledge, very few studies have investigated the causal mechanisms of these symptoms for either of these two viruses. One study [110] found IAPV particles to accumulate mostly in the gut, hypopharyngeal glands and nerve cells. Aggregation in the hypopharyngeal glands can be strategic for the virus to spread from one host to another, but aggregation in nerves suggests potential neural alterations causing trembling and paralysis. In contrast, other authors [218] have found viral particles to accumulate mostly in the fat body and trachea. Evidence of mitochondrial respiratory disruption in tracheal cells caused by the viral invasion may be the actual cause of the symptoms and abrupt death following AKI infection. This hypothesis needs to be further investigated to determine whether other cell types are affected in this way and whether CBPV inflicts similar disruption in other types of invaded cells. Such investigations may provide hints as to potential interactions between these two viruses and whether they are competitive or synergistic in nature.

## 4. Perspectives

Immune responses are inherently costly and likely reduce fitness. The immune challenge following heat-killed bacteria inoculation results in reduced foraging efficiency in bumblebees [219,220,221] and reduced lifespan in both bumblebees and honey bees [222]. As discussed in [164], it is to the advantage of the bee to allocate resources to the most effective immune pathways considering current threats. This plasticity in immune responses may reflect the need to minimise the fitness costs of such responses [223].

Here, we described the activation of different immune pathways following DWV infection depending on the developmental stage of the bee. As the immune response can change through the lifetime of the bee, the observed immune gene expression following infection may be representative of this age-related variation in the bee immune response. However, in these studies, all artificial DWV inoculations at the larval stage have been conducted through the oral route, but at later developmental stages, the analysed bees are either naturally infected or inoculated through direct injection. Different administration routes may trigger different immune and viral dynamics. Indeed, changes in rates of replication and virulence have been found for BQCV [9,224] and SBV [9] between cases of oral inoculations and direct injections. In another arthropod (*Armadillidium vulgare*), a common endosymbiont (*Wolbachia*) has been shown to become pathogenic when a shift from vertical to horizontal transmission occurs [225]. In all these cases, the change in transmission route made the microbe more virulent and increased its replication. As discussed in Section 3.1, a similar pattern was observed when DWV became vectored by varroa, with differential immune gene expression between orally inoculated and varroa-mediated inoculated bees [87].

We described two viruses triggering the inhibition of the Toll pathway upon infection in adult bees: DWV and IAPV (see Figure 2). Some studies in other insects suggest that the Jak-STAT pathway is activated following Dicer-2 activity through *vago* expression [139,140]. The Jak-STAT pathway is known to be involved in the regulation of other immune pathways, such as Toll [226]. As both DWV and IAPV trigger Dicer-2 activity and activation of the Jak-STAT pathway, the observed Toll pathway inhibition following infection may be an integral part of the immune strategy of the bee, reallocating resources to RNAi, an immune response specifically targeting viruses. If honey bees indeed deploy such a cascade of immune regulations, and these viruses indeed evade RNAi effectors while still triggering Dicer-2 activity, the RNAi and the humoral pathways together would be mostly ineffective, leaving the honey bee defenceless against their replication. Additionally, both viruses may replicate within varroa mites and be transmitted by them [96,98], also favouring their replication. However, although Jak-STAT’s Domeless receptor is involved in the Toll pathway inhibition, it has been suggested that the Vago protein does not interact with this specific receptor, suggesting an alternative Jak-STAT activation. Nonetheless, any putative crosstalk between different immune pathways must be further investigated to understand the honey bee immune response as a dynamical, coordinated, and complex system.

Similarly, the RNAi immune pathway of bees infected with a high number of different viruses can be disrupted, as opposed to honey bees infected with only a few different viruses [227]. This latter study focuses on the depletion of the immune response. However, an alternative interpretation is that multiple co-infections induce a shift in bee immune strategy, not unlike high predatory stress or nutritional stress can induce reconfigurations in immune responses [228,229]. As opposed to predatory or nutritional stress, a shift in immune strategy driven by multiple co-infections may not be in favour of reinforcement of primary defences. In this case, the immune system would more likely reallocate resources to pathways involved in long-term immune strategies or generalist ones. The production of AMPs, being known as a more generalist immune response [47], would be in line with the depletion of the RNAi pathway and the parallel increase in AMPs revealed in this study [227]. The RNAi core machinery is driven by specific virus targeting, but the humoral immune pathways may be effective against all major honey bee viruses, perhaps driving a shift in immune strategy when facing multiple co-infections.

As we saw in Section 3.2, DWV and SBV interactions seem to be the most beneficial to SBV. Although DWV infections seem to indirectly help SBV replication, the increased virulence following SBV replication may not be in favour of DWV transmission. Both viruses can be transmitted vertically [230], but it is the relatively recent change in the DWV route of transmission via varroa that has notably increased promiscuity—and thus potential interactions—between these two viruses [231], although the decrease in hygiene behaviour observed in DWV-infected individuals (see Section 2.5) might decrease co-infection occurrences. In Asian countries where varroa has been established for longer times, Chinese SBV (CSBV) has become a major threat to Asian honey bees (*A. cerana*) [101,102,232]. Therefore, we should carefully monitor the evolution of western strains of SBV because they may become more prevalent and more virulent through their interactions with DWV. Fortunately, recent independent efforts have led to the elaboration of infectious viral clones containing GFP for both DWV [233] and SBV [234]. These advances will surely help future research in enhancing our understanding of infection and co-infection mechanisms between these two viruses to help predict their future evolution and manage them effectively.

## Data Availability

No data was created for this study.

## References

[1] Klein A.M., Vaissière B.E., Cane J.H., Steffan-Dewenter I., Cunningham S.A., Kremen C., Tscharntke T. (2007). Importance of Pollinators in Changing Landscapes for World Crops. Proc. R. Soc. B Biol. Sci..

[2] Khalifa S.A.M., Elshafiey E.H., Shetaia A.A., El-Wahed A.A.A., Algethami A.F., Musharraf S.G., AlAjmi M.F., Zhao C., Masry S.H.D., Abdel-Daim M.M. (2021). Overview of bee pollination and its economic value for crop production. Insects.

[3] Goulson D., Nicholls E., Botías C., Rotheray E.L. (2015). Bee Declines Driven by Combined Stress from Parasites, Pesticides, and Lack of Flowers. Science.

[4] Coulon M., Dalmon A., Di Prisco G., Prado A., Arban F., Dubois E., Ribière-Chabert M., Alaux C., Thiéry R., Le Conte Y. (2020). Interactions Between Thiamethoxam and Deformed Wing Virus Can Drastically Impair Flight Behavior of Honey Bees. Front. Microbiol..

[5] O’Neal S.T., Reeves A.M., Fell R.D., Brewster C.C., Anderson T.D. (2019). Chlorothalonil Exposure Alters Virus Susceptibility and Markers of Immunity, Nutrition, and Development in Honey Bees. J. Insect Sci..

[6] Cohen H., Smith G.P., Sardiñas H., Zorn J.F., McFrederick Q.S., Woodard S.H., Ponisio L.C. (2021). Mass-Flowering Monoculture Attracts Bees, Amplifying Parasite Prevalence. Proc. R. Soc. B Boil. Sci..

[7] Gisder S., Aumeier P., Genersch E. (2009). Deformed Wing Virus: Replication and Viral Load in Mites (*Varroa destructor*). J. Gen. Virol..

[8] Hedtke K., Jensen P.M., Jensen A.B., Genersch E. (2011). Evidence for Emerging Parasites and Pathogens Influencing Outbreaks of Stress-Related Diseases like Chalkbrood. J. Invertebr. Pathol..

[9] Remnant E.J., Mather N., Gillard T.L., Yagound B., Beekman M. (2019). Direct Transmission by Injection Affects Competition among RNA Viruses in Honeybees. Proc. R. Soc. B Boil. Sci..

[10] Tosi S., Nieh J.C., Sgolastra F., Cabbri R., Medrzycki P. (2017). Neonicotinoid Pesticides and Nutritional Stress Synergistically Reduce Survival in Honey Bees. Proc. R. Soc. B Boil. Sci..

[11] DeGrandi-Hoffman G., Chen Y. (2015). Nutrition, Immunity and Viral Infections in Honey Bees. Curr. Opin. Insect Sci..

[12] Tritschler M., Vollmann J.J., Yañez O., Chejanovsky N., Crailsheim K., Neumann P. (2017). Protein Nutrition Governs within-Host Race of Honey Bee Pathogens. Sci. Rep..

[13] Zheng H.Q., Gong H.R., Huang S.K., Sohr A., Hu F.L., Chen Y.P. (2015). Evidence of the Synergistic Interaction of Honey Bee Pathogens *Nosema ceranae* and *Deformed wing virus*. Vet. Microbiol..

[14] Gajda A.M., Mazur E.D., Bober A.M., Czopowicz M. (2021). *Nosema ceranae* Interactions with *Nosema apis* and Black Queen Cell Virus. Agriculture.

[15] El-Seedi H.R., Ahmed H.R., El-Wahed A.A.A., Saeed A., Algethami A.F., Attia N.F., Guo Z., Musharraf S.G., Khatib A., Alsharif S.M. (2022). Bee stressors from an immunological perspective and strategies to improve bee health. Vet. Sci..

[16] Beaurepaire A., Piot N., Doublet V., Antunez K., Campbell E., Chantawannakul P., Chejanovsky N., Gajda A., Heerman M., Panziera D. (2020). Diversity and Global Distribution of Viruses of the Western Honey Bee, *Apis mellifera*. Insects.

[17] Méthot P.O., Alizon S. (2014). What Is a Pathogen? Toward a Process View of Host-Parasite Interactions. Virulence.

[18] Casadevall A., Pirofski L.A. (2003). The Damage-Response Framework of Microbial Pathogenesis. Nat. Rev. Microbiol..

[19] Casadevall A., Pirofski L.A. (1999). Host-Pathogen Interactions: Redefining the Basic Concepts of Virulence and Pathogenicity. Infect. Immun..

[20] Hosokawa T., Ishii Y., Nikoh N., Fujie M., Satoh N., Fukatsu T. (2016). Obligate Bacterial Mutualists Evolving from Environmental Bacteria in Natural Insect Populations. Nat. Microbiol..

[21] Roossinck M.J. (2011). The Good Viruses: Viral Mutualistic Symbioses. Nat. Rev. Microbiol..

[22] Belden L.K., Harris R.N. (2007). Infectious Diseases in Wildlife: The Community Ecology Context. Front. Ecol. Environ..

[23] Daskin J.H., Alford R.A. (2012). Context-Dependent Symbioses and Their Potential Roles in Wildlife Diseases. Proc. R. Soc. B Boil. Sci..

[24] Hajishengallis G., Lamont R.J. (2016). Dancing with the Stars: How Choreographed Bacterial Interactions Dictate Nososymbiocity and Give Rise to Keystone Pathogens, Accessory Pathogens, and Pathobionts. Trends Microbiol..

[25] Schurr F., Tison A., Militano L., Cheviron N., Sircoulomb F., Rivière M.P., Ribière-Chabert M., Thiéry R., Dubois E. (2019). Validation of Quantitative Real-Time RT-PCR Assays for the Detection of Six Honeybee Viruses. J. Virol. Methods.

[26] De Miranda J.R., Genersch E. (2010). Deformed Wing Virus. J. Invertebr. Pathol..

[27] Cooper D., Cory J.S., Theilmann D.A., Myers J.H. (2003). Nucleopolyhedroviruses of Forest and Western Tent Caterpillars: Cross-Infectivity and Evidence for Activation of Latent Virus in High-Density Field Populations. Ecol. Entomol..

[28] Boots M., Greenman J., Ross D., Norman R., Hails R., Sait S. (2003). The Population Dynamical Implications of Covert Infections in Host-Microparasite Interactions. J. Anim. Ecol..

[29] Bailey L., Gibbs A.J., Woods R.D. (1964). Sacbrood Virus of the Larval Honey Bee (*Apis mellifera* Linnaeus). Virology.

[30] Wei R., Cao L., Feng Y., Chen Y., Chen G., Zheng H. (2022). Sacbrood Virus: A Growing Threat to Honeybees and Wild Pollinators. Viruses.

[31] Bailey L., Woods R.D. (1977). Two More Small RNA Viruses from Honey Bees and Further Observations on Sacbrood and Acute Bee-Paralysis Viruses. J. Gen. Virol..

[32] Spurny R., Pridal A. (2017). Virion Structure of Black Queen Cell Virus, a Common Honeybee Pathogen. J. Virol..

[33] Paxton R.J., Schäfer M.O., Nazzi F., Zanni V., Annoscia D., Marroni F., Bigot D., Laws-Quinn E.R., Panziera D., Jenkins C. (2022). Epidemiology of a Major Honey Bee Pathogen, Deformed Wing Virus: Potential Worldwide Replacement of Genotype A by Genotype B. Int. J. Parasitol. Parasites Wildl..

[34] Bailey L. (1965). Paralysis of the Honey Bee, *Apis mellifera* Linnaeus. J. Invertebr. Pathol..

[35] Ribière M., Olivier V., Blanchard P. (2010). Chronic Bee Paralysis: A Disease and a Virus like No Other?. J. Invertebr. Pathol..

[36] Bailey L., Gibbs A.J., Woods R.D. (1963). Two Viruses from Adult Honey Bees (*Apis mellifera* Linnaeus). Virology.

[37] De Miranda J.R., Cordoni G., Budge G. (2010). The Acute Bee Paralysis Virus-Kashmir Bee Virus-Israeli Acute Paralysis Virus Complex. J. Invertebr. Pathol..

[38] Maebe K., Vereecken N.J., Piot N., Reverté S., Cejas D., Michez D., Vandamme P., Smagghe G. (2021). The Holobiont as a Key to the Adaptation and Conservation of Wild Bees in the Anthropocene. Front. Ecol. Evol..

[39] Bordenstein S.R., Theis K.R. (2015). Host Biology in Light of the Microbiome: Ten Principles of Holobionts and Hologenomes. PLoS Biol..

[40] Theis K.R., Dheilly N.M., Klassen J.L., Brucker R.M., Baines J.F., Bosch T.C.G., Cryan J.F., Gilbert S.F., Goodnight C.J., Lloyd E.A. (2016). Getting the Hologenome Concept Right: An Eco-Evolutionary Framework for Hosts and Their Microbiomes. Msystems.

[41] Schwarz R.S., Huang Q., Evans J.D. (2015). Hologenome Theory and the Honey Bee Pathosphere. Curr. Opin. Insect Sci..

[42] Tecon R., Mitri S., Ciccarese D., Or D., van der Meer J.R., Johnson D.R. (2019). Bridging the Holistic-Reductionist Divide in Microbial Ecology. mSystems.

[43] Fang F.C., Casadevall A. (2011). Reductionistic and Holistic Science. Infect. Immun..

[44] Brutscher L.M., Daughenbaugh K.F., Flenniken M.L. (2015). Antiviral Defense Mechanisms in Honey Bees. Curr. Opin. Insect Sci..

[45] Doublet V., Poeschl Y., Gogol-Döring A., Alaux C., Annoscia D., Aurori C., Barribeau S.M., Bedoya-Reina O.C., Brown M.J.F., Bull J.C. (2017). Unity in Defence: Honeybee Workers Exhibit Conserved Molecular Responses to Diverse Pathogens. BMC Genom..

[46] Negri P., Maggi M., Ramirez L., Szawarski N., De Feudis L., Lamattina L., Eguaras M. (2016). Cellular Immunity in *Apis mellifera*: Studying Hemocytes Brings Light about Bees Skills to Confront Threats. Apidologie.

[47] Simone-Finstrom M. (2017). Social Immunity and the Superorganism: Behavioural Defenses Protecting Honey Bee Colonies from Pathogens and Parasites. Bee World.

[48] Cohen A.A., Martin L.B., Wingfield J.C., McWilliams S.R., Dunne J.A. (2012). Physiological Regulatory Networks: Ecological Roles and Evolutionary Constraints. Trends Ecol. Evol..

[49] Lemaitre B., Hoffmann J. (2007). The host defense of Drosophila melanogaster. Annu. Rev. Immunol..

[50] Kumar A., Srivastava P., Sirisena P.D.N.N., Dubey S.K., Kumar R., Shrinet J., Sunil S. (2018). Mosquito innate immunity. Insects.

[51] Mondet F., Alaux C., Severac D., Rohmer M., Mercer A.R., Le Conte Y. (2015). Antennae Hold a Key to Varroa-Sensitive Hygiene Behaviour in Honey Bees. Sci. Rep..

[52] Mondet F., Kim S.H., De Miranda J.R., Beslay D., Le Conte Y., Mercer A.R. (2016). Specific Cues Associated with Honey Bee Social Defence against *Varroa destructor* Infested Brood. Sci. Rep..

[53] Vung N.N., Choi Y.S., Kim I. (2020). High Resistance to Sacbrood Virus Disease in *Apis cerana* (Hymenoptera: Apidae) Colonies Selected for Superior Brood Viability and Hygienic Behavior. Apidologie.

[54] Schöning C., Gisder S., Geiselhardt S., Kretschmann I., Bienefeld K., Hilker M., Genersch E. (2012). Evidence for Damage-Dependent Hygienic Behaviour towards *Varroa destructor*-Parasitised Brood in the Western Honey Bee, *Apis mellifera*. J. Exp. Biol..

[55] Shah K.S., Evans E.C., Pizzorno M.C. (2009). Localization of Deformed Wing Virus (DWV) in the Brains of the Honeybee, *Apis mellifera* Linnaeus. Virol. J..

[56] Lin Z., Page P., Li L., Qin Y., Zhang Y., Hu F., Neumann P., Zheng H., Dietemann V. (2016). Go East for Better Honey Bee Health: *Apis cerana* Is Faster at Hygienic Behavior than *A. mellifera*. PLoS ONE.

[57] Rinderer T.E., Rothenbuhler W.C. (1976). Characteristic Field Symptoms Comprising Honeybee Hairless-Black Syndrome Induced in the Laboratory by a Virus. J. Invertebr. Pathol..

[58] Richard F.J., Aubert A., Grozinger C.M. (2008). Modulation of Social Interactions by Immune Stimulation in Honey Bee, *Apis mellifera*, Workers. BMC Biol..

[59] Baracchi D., Fadda A., Turillazzi S. (2012). Evidence for Antiseptic Behaviour towards Sick Adult Bees in Honey Bee Colonies. J. Insect Physiol..

[60] Geffre A.C., Gernat T., Harwood G.P., Jones B.M., Gysi D.M., Hamilton A.R., Bonning B.C., Toth A.L., Robinson G.E., Dolezal A.G. (2020). Honey Bee Virus Causes Context-Dependent Changes in Host Social Behavior. Proc. Natl. Acad. Sci. USA.

[61] Amiri E., Seddon G., Smith W.Z., Strand M.K., Tarpy D.R., Rueppell O. (2019). Israeli acute paralysis virus: Honey bee queen–worker interaction and potential virus transmission pathways. Insects.

[62] Salmela H., Amdam G.V., Freitak D. (2015). Transfer of Immunity from Mother to Offspring Is Mediated via Egg-Yolk Protein Vitellogenin. PLoS Pathog..

[63] Maori E., Garbian Y., Kunik V., Mozes-Koch R., Malka O., Kalev H., Sabath N., Sela I., Shafir S. (2019). A Transmissible RNA Pathway in Honey Bees. Cell Rep..

[64] López J.H., Schuehly W., Crailsheim K., Riessberger-Gallé U. (2014). Trans-Generational Immune Priming in Honeybees. Proc. R. Soc. B Biol. Sci..

[65] De Graaf D.C., Laget D., De Smet L., Claeys Boúúaert D., Brunain M., Veerkamp R.F., Brascamp E.W. (2020). Heritability Estimates of the Novel Trait ‘Suppressed in Ovo Virus Infection’ in Honey Bees (*Apis mellifera*). Sci. Rep..

[66] Leponiemi M., Amdam G.V., Freitak D. (2021). Exposure to Inactivated Deformed Wing Virus Leads to Trans-Generational Costs but Not Immune Priming in Honeybees (*Apis mellifera*). Front. Ecol. Evol..

[67] Lang S., Simone-Finstrom M., Healy K. (2022). Context-Dependent Viral Transgenerational Immune Priming in Honey Bees (Hymenoptera: Apidae). J. Insect Sci..

[68] Saelao P., Borba R.S., Ricigliano V., Spivak M., Simone-Finstrom M. (2020). Honeybee microbiome is stabilized in the presence of propolis. Biol. Lett..

[69] Ramsey S.D., Ochoa R., Bauchan G., Gulbronson C., Mowery J.D., Cohen A., Lim D., Joklik J., Cicero J.M., Ellis J.D. (2019). *Varroa destructor* Feeds Primarily on Honey Bee Fat Body Tissue and Not Hemolymph. Proc. Natl. Acad. Sci. USA.

[70] Aronstein K.A., Saldivar E., Vega R., Westmiller S., Douglas A.E. (2012). How *Varroa* Parasitism Affects the Immunological and Nutritional Status of the Honey Bee, *Apis mellifera*. Insects.

[71] Annoscia D., Brown S.P., Di Prisco G., De Paoli E., Del Fabbro S., Frizzera D., Zanni V., Galbraith D.A., Caprio E., Grozinger C.M. (2019). Haemolymph Removal by Varroa Mite Destabilizes the Dynamical Interaction between Immune Effectors and Virus in Bees, as Predicted by Volterra’s Model. Proc. R. Soc. B Biol. Sci..

[72] Alaux C., Ducloz F., Crauser D., Le Conte Y. (2010). Diet Effects on Honeybee Immunocompetence. Biol. Lett..

[73] Billiet A., Meeus I., Van Nieuwerburgh F., Deforce D., Wäckers F., Smagghe G. (2016). Impact of sugar syrup and pollen diet on the bacterial diversity in the gut of indoor-reared bumblebees (*Bombus terrestris*). Apidologie.

[74] Maes P.W., Rodrigues P.A.P., Oliver R., Mott B.M., Anderson K.E. (2016). Diet-related gut bacterial dysbiosis correlates with impaired development, increased mortality and Nosema disease in the honeybee (*Apis mellifera*). Mol. Ecol..

[75] Haag K.L., Caesar L., da Silveira Regueira-Neto M., de Sousa D.R., Montenegro Marcelino V., de Queiroz Balbino V., Torres Carvalho A. (2022). Temporal Changes in Gut Microbiota Composition and Pollen Diet Associated with Colony Weakness of a Stingless Bee. Microb. Ecol..

[76] Steele M.I., Motta E.V.S., Gattu T., Martinez D., Moran N.A. (2021). The Gut Microbiota Protects Bees from Invasion by a Bacterial Pathogen. Microbiol. Spectr..

[77] Horak R.D., Leonard S.P., Moran N.A. (2020). Symbionts Shape Host Innate Immunity in Honeybees: Symbionts Shape Honey Bee Immunity. Proc. R. Soc. B Biol. Sci..

[78] Raymann K., Moran N.A. (2018). The role of the gut microbiome in health and disease of adult honey bee workers. Curr. Opin. Insect Sci..

[79] Deboutte W., Beller L., Yinda C.K., Maes P., de Graaf D.C., Matthijnssens J. (2020). Honey-bee–associated prokaryotic viral communities reveal wide viral diversity and a profound metabolic coding potential. Proc. Natl. Acad. Sci. USA.

[80] Dosch C., Manigk A., Streicher T., Tehel A., Paxton R.J., Tragust S. (2021). The Gut Microbiota Can Provide Viral Tolerance in the Honey Bee. Microorganisms.

[81] Kim C., Kim J.M., Choi H., Choi Y.S., Jin B.R., Lee K.S., Choi K. (2022). Analysis of the gut microbiome of susceptible and resistant honeybees (*Apis cerana*) against sacbrood virus disease. J. Appl. Entomol..

[82] Yun B.R., Truong A.T., Choi Y.S., Lee M.Y., Kim B.Y., Seo M., Yoon S.S., Yoo M.S., Van Quyen D., Cho Y.S. (2022). Comparison of the gut microbiome of sacbrood virus-resistant and -susceptible *Apis cerana* from South Korea. Sci. Rep..

[83] Raymann K., Shaffer Z., Moran N.A. (2017). Antibiotic exposure perturbs the gut microbiota and elevates mortality in honeybees. PLoS Biol..

[84] Deng Y., Yang S., Zhao H., Luo J., Yang W., Hou C. (2022). Antibiotics-induced changes in intestinal bacteria result in the sensitivity of honey bee to virus. Environ. Pollut..

[85] Bazin T., Chiu L., Pradeu T. (2022). Host-Microbiota Co-Immunity: An Intimate Relationship That Goes Beyond Protection. Philos. Theory Pract. Biol..

[86] Chiu L., Bazin T., Truchetet M.E., Schaeverbeke T., Delhaes L., Pradeu T. (2017). Protective Microbiota: From Localized to Long-Reaching Co-Immunity. Front. Immunol..

[87] Ryabov E.V., Wood G.R., Fannon J.M., Moore J.D., Bull J.C., Chandler D., Mead A., Burroughs N., Evans D.J. (2014). A Virulent Strain of Deformed Wing Virus (DWV) of Honeybees (*Apis mellifera*) Prevails after *Varroa destructor*-Mediated, or In Vitro, Transmission. PLoS Pathog..

[88] Dalmon A., Desbiez C., Coulon M., Thomasson M., Le Conte Y., Alaux C., Vallon J., Moury B. (2017). Evidence for Positive Selection and Recombination Hotspots in Deformed Wing Virus (DWV). Sci. Rep..

[89] Levin S., Sela N., Erez T., Nestel D., Pettis J., Neumann P., Chejanovsky N. (2019). New Viruses from the Ectoparasite Mite *Varroa destructor* Infesting *Apis mellifera* and *Apis cerana*. Viruses.

[90] Posada-Florez F., Ryabov E.V., Heerman M.C., Chen Y., Evans J.D., Sonenshine D.E., Cook S.C. (2020). *Varroa destructor* Mites Vector and Transmit Pathogenic Honey Bee Viruses Acquired from an Artificial Diet. PLoS ONE.

[91] Martin S.J., Highfield A.C., Brettell L., Villalobos E.M., Budge G.E., Powell M., Nikaido S., Schroeder D.C. (2012). Global Honey Bee Viral Landscape Altered by a Parasitic Mite. Science.

[92] Ray A.M., Davis S.L., Rasgon J.L., Grozinger C.M. (2021). Simulated Vector Transmission Differentially Influences Dynamics of Two Viral Variants of Deformed Wing Virus in Honey Bees (*Apis mellifera*). J. Gen. Virol..

[93] Piou V., Schurr F., Dubois E., Vétillard A. (2022). Transmission of Deformed Wing Virus between *Varroa destructor* Foundresses, Mite Offspring and Infested Honey Bees. Parasites Vectors.

[94] Ryabov E.V., Childers A.K., Lopez D., Grubbs K., Posada-Florez F., Weaver D., Girten W., van Engelsdorp D., Chen Y., Evans J.D. (2019). Dynamic Evolution in the Key Honey Bee Pathogen Deformed Wing Virus: Novel Insights into Virulence and Competition Using Reverse Genetics. PLoS Biol..

[95] Ongus J.R., Peters D., Bonmatin J.M., Bengsch E., Vlak J.M., van Oers M.M. (2004). Complete Sequence of a Picorna-like Virus of the Genus *Iflavirus* Replicating in the Mite *Varroa destructor*. J. Gen. Virol..

[96] Gisder S., Genersch E. (2021). Direct Evidence for Infection of *Varroa destructor* Mites with the Bee-Pathogenic Deformed Wing Virus Variant B, but Not Variant A, via Fluorescence In Situ Hybridization Analysis. J. Virol..

[97] Moore J., Jironkin A., Chandler D., Burroughs N., Evans D.J., Ryabov E.V. (2011). Recombinants between Deformed Wing Virus and *Varroa destructor* Virus-1 May Prevail in *Varroa destructor*-Infested Honeybee Colonies. J. Gen. Virol..

[98] Di Prisco G., Pennacchio F., Caprio E., Boncristiani H.F., Evans J.D., Chen Y. (2011). *Varroa destructor* is an effective vector of Israeli acute paralysis virus in the honeybee, *Apis mellifera*. J. Gen. Virol..

[99] Ryabov E.V., Fannon J.M., Moore J.D., Wood G.R., Evans D.J. (2016). The Iflaviruses Sacbrood Virus and Deformed Wing Virus Evoke Different Transcriptional Responses in the Honeybee Which May Facilitate Their Horizontal or Vertical Transmission. PeerJ.

[100] Shan L., Liuhao W., Jun G., Yujie T., Yanping C., Jie W., Jilian L. (2017). Chinese Sacbrood Virus Infection in Asian Honey Bees (*Apis cerana cerana*) and Host Immune Responses to the Virus Infection. J. Invertebr. Pathol..

[101] Zhang Y., Huang X., Xu Z., Han R., Chen J. (2013). Differential Gene Transcription in Honeybee (*Apis cerana*) Larvae Challenged by Chinese Sacbrood Virus (CSBV). Sociobiology.

[102] Guo Y., Zhang Z., Zhuang M., Wang L., Li K., Yao J., Yang H., Huang J., Hao Y., Ying F. (2021). Transcriptome Profiling Reveals a Novel Mechanism of Antiviral Immunity Upon Sacbrood Virus Infection in Honey Bee Larvae (*Apis cerana*). Front. Microbiol..

[103] Quintana S., Brasesco C., Negri P., Marin M., Pagnuco I., Szawarski N., Reynaldi F.J., Larsen A., Eguaras M., Maggi M. (2019). Up-Regulated Pathways in Response to Deformed Wing Virus Infection in *Apis mellifera* (Hymenoptera: Apidae). Rev. Soc. Entomol. Argent..

[104] Yang X., Cox-Foster D.L. (2005). Impact of an Ectoparasite on the Immunity and Pathology of an Invertebrate: Evidence for Host Immunosuppression and Viral Amplification. Proc. Natl. Acad. Sci. USA.

[105] Di Prisco G., Annoscia D., Margiotta M., Ferrara R., Varricchio P., Zanni V., Caprio E., Nazzi F., Pennacchio F. (2016). A Mutualistic Symbiosis between a Parasitic Mite and a Pathogenic Virus Undermines Honey Bee Immunity and Health. Proc. Natl. Acad. Sci. USA.

[106] Barroso-Arévalo S., Vicente-Rubiano M., Puerta F., Molero F., Sánchez-Vizcaíno J.M. (2019). Immune Related Genes as Markers for Monitoring Health Status of Honey Bee Colonies. BMC Vet. Res..

[107] Mookhploy W., Krongdang S., Chantawannakul P. (2021). Effects of Deformed Wing Virus Infection on Expressions of Immune-and Apoptosis-Related Genes in Western Honeybees (*Apis mellifera*). Insects.

[108] Nazzi F., Brown S.P., Annoscia D., Del Piccolo F., Di Prisco G., Varricchio P., Della Vedova G., Cattonaro F., Caprio E., Pennacchio F. (2012). Synergistic Parasite-Pathogen Interactions Mediated by Host Immunity Can Drive the Collapse of Honeybee Colonies. PLoS Pathog..

[109] Zhao Y., Heerman M., Peng W., Evans J.D., Rose R., Degrandi-Hoffman G., Simone-Finstrom M., Li J., Li Z., Cook S.C. (2019). The Dynamics of Deformed Wing Virus Concentration and Host Defensive Gene Expression after Varroa Mite Parasitism in Honey Bees, *Apis mellifera*. Insects.

[110] Chen Y.P., Pettis J.S., Corona M., Chen W.P., Li C.J., Spivak M., Visscher P.K., DeGrandi-Hoffman G., Boncristiani H., Zhao Y. (2014). Israeli Acute Paralysis Virus: Epidemiology, Pathogenesis and Implications for Honey Bee Health. PLoS Pathog..

[111] Chejanovsky N., Ophir R., Schwager M.S., Slabezki Y., Grossman S., Cox-Foster D. (2014). Characterization of Viral SiRNA Populations in Honey Bee Colony Collapse Disorder. Virology.

[112] Galbraith D.A., Yang X., Niño E.L., Yi S., Grozinger C. (2015). Parallel Epigenomic and Transcriptomic Responses to Viral Infection in Honey Bees (*Apis mellifera*). PLoS Pathog..

[113] Azzami K., Ritter W., Tautz J., Beier H. (2012). Infection of Honey Bees with Acute Bee Paralysis Virus Does Not Trigger Humoral or Cellular Immune Responses. Arch. Virol..

[114] Hayward A., Takahashi T., Bendena W.G., Tobe S.S., Hui J.H.L. (2010). Comparative Genomic and Phylogenetic Analysis of Vitellogenin and Other Large Lipid Transfer Proteins in Metazoans. FEBS Lett..

[115] Hagedorn H.H., Kunkel J.G. (1979). Vitellogenin and Vitellin in Insects. Annu. Rev. Entomol..

[116] Amdam G.V., Norberg K., Hagen A., Omholt S.W. (2003). Social Exploitation of Vitellogenin. Proc. Natl. Acad. Sci. USA.

[117] Amdam G.V., Omholt S.W. (2003). The Hive Bee to Forager Transition in Honeybee Colonies: The Double Repressor Hypothesis. J. Theor. Biol..

[118] Seehuus S.C., Norberg K., Krekling T., Fondrk K., Amdam G.V. (2007). Immunogold Localization of Vitellogenin in the Ovaries, Hypopharyngeal Glands and Head Fat Bodies of Honeybee Workers, *Apis mellifera*. J. Insect Sci..

[119] Fluri P., Lüscher M., Wille H., Gerig L. (1982). Changes in Weight of the Pharyngeal Gland and Haemolymph Titres of Juvenile Hormone, Protein and Vitellogenin in Worker Honey Bees. J. Insect Physiol..

[120] Pinto L.Z., Bitondi M.M.G., Simões Z.L.P. (2000). Inhibition of Vitellogenin Synthesis in *Apis Mellifera* Workers by a Juvenile Hormone Analogue, Pyriproxyfen. J. Insect Physiol..

[121] Piulachs M.D., Guidugli K.R., Barchuk A.R., Cruz J., Simões Z.L.P., Bellés X. (2003). The Vitellogenin of the Honey Bee, *Apis mellifera*: Structural Analysis of the CDNA and Expression Studies. Insect Biochem. Mol. Biol..

[122] Amdam G.V., Simões Z.L.P., Hagen A., Norberg K., Schrøder K., Mikkelsen Ø., Kirkwood T.B.L., Omholt S.W. (2004). Hormonal Control of the Yolk Precursor Vitellogenin Regulates Immune Function and Longevity in Honeybees. Exp. Gerontol..

[123] Amdam G.V., Norberg K., Page R.E., Erber J., Scheiner R. (2006). Downregulation of Vitellogenin Gene Activity Increases the Gustatory Responsiveness of Honey Bee Workers (*Apis mellifera*). Behav. Brain Res..

[124] Amdam G.V., Aase A.L.T.O., Seehuus S.C., Kim Fondrk M., Norberg K., Hartfelder K. (2005). Social Reversal of Immunosenescence in Honey Bee Workers. Exp. Gerontol..

[125] Seehuus S.C., Norberg K., Gimsa U., Krekling T., Amdam G.V. (2006). Reproductive Protein Protects Functionally Sterile Honey Bee Workers from Oxidative Stress. Proc. Natl. Acad. Sci. USA.

[126] Bordier C., Suchail S., Pioz M., Devaud J.M., Collet C., Charreton M., Le Conte Y., Alaux C. (2017). Stress Response in Honeybees Is Associated with Changes in Task-Related Physiology and Energetic Metabolism. J. Insect Physiol..

[127] Prado A., Brunet J.L., Peruzzi M., Bonnet M., Bordier C., Crauser D., Le Conte Y., Alaux C. (2022). Warmer Winters Are Associated with Lower Levels of the Cryoprotectant Glycerol, a Slower Decrease in Vitellogenin Expression and Reduced Virus Infections in Winter Honeybees. J. Insect Physiol..

[128] Dalmon A., Peruzzi M., Le Conte Y., Alaux C., Pioz M. (2019). Temperature-Driven Changes in Viral Loads in the Honey Bee *Apis mellifera*. J. Invertebr. Pathol..

[129] Lin Y.W., Chen C.H., Hsu C.Y. (2022). Middle-Aged Worker Bees Express Higher Innate Immunity than Young Worker Bees in the Abdomen without the Digestive Tract of Worker Bees Reared in an Incubator. Insects.

[130] Bull J.C., Ryabov E.V., Prince G., Mead A., Zhang C., Baxter L.A., Pell J.K., Osborne J.L., Chandler D. (2012). A Strong Immune Response in Young Adult Honeybees Masks Their Increased Susceptibility to Infection Compared to Older Bees. PLoS Pathog..

[131] Steinmann N., Corona M., Neumann P., Dainat B. (2015). Overwintering Is Associated with Reduced Expression of Immune Genes and Higher Susceptibility to Virus Infection in Honey Bees. PLoS ONE.

[132] Brutscher L.M., Daughenbaugh K.F., Flenniken M.L. (2017). Virus and DsRNA-Triggered Transcriptional Responses Reveal Key Components of Honey Bee Antiviral Defense. Sci. Rep..

[133] Hammond S.M. (2005). Dicing and Slicing: The Core Machinery of the RNA Interference Pathway. FEBS Lett..

[134] Schuster S., Miesen P., van Rij R.P. (2019). Antiviral RNAi in insects and mammals: Parallels and differences. Viruses.

[135] Shabalina S.A., Koonin E. (2008). V Origins and Evolution of Eukaryotic RNA Interference the MiRNA and SiRNA Machinery. Trends Ecol. Evol..

[136] Torri A., Jaeger J., Pradeu T., Saleh M.C. (2022). The Origin of RNA Interference: Adaptive or Neutral Evolution?. PLoS Biol..

[137] Zamore P.D., Haley B. (2005). Ribo-Gnome: The Big World of Small RNAs. Science.

[138] Neumeier J., Meister G. (2021). SiRNA Specificity: RNAi Mechanisms and Strategies to Reduce Off-Target Effects. Front. Plant Sci..

[139] Deddouche S., Matt N., Budd A., Mueller S., Kemp C., Galiana-Arnoux D., Dostert C., Antoniewski C., Hoffmann J.A., Imler J.L. (2008). The DExD/H-Box Helicase Dicer-2 Mediates the Induction of Antiviral Activity in Drosophila. Nat. Immunol..

[140] Paradkar P.N., Trinidad L., Voysey R., Duchemin J.B., Walker P.J. (2012). Secreted Vago Restricts West Nile Virus Infection in Culex Mosquito Cells by Activating the Jak-STAT Pathway. Proc. Natl. Acad. Sci. USA.

[141] Bang I.S. (2019). INVI TED R EVI EW JAK/STAT Signaling in Insect Innate Immunity. Entomol. Res..

[142] Merkling S.H., van Rij R.P. (2013). Beyond RNAi: Antiviral defense strategies in Drosophila and mosquito. J. Insect Physiol..

[143] Schlüns H., Crozier R.H. (2007). Relish Regulates Expression of Antimicrobial Peptide Genes in the Honeybee, *Apis mellifera*, Shown by RNA Interference. Insect Mol. Biol..

[144] Evans J.D., Aronstein K., Chen Y.P., Hetru C., Imler J.L., Jiang H., Kanost M., Thompson G.J., Zou Z., Hultmark D. (2006). Immune Pathways and Defence Mechanisms in Honey Bees *Apis mellifera*. Insect Mol. Biol..

[145] Nishide Y., Kageyama D., Yokoi K., Jouraku A., Tanaka H., Futahashi R., Fukatsu T. (2019). Functional Crosstalk across IMD and Toll Pathways: Insight into the Evolution of Incomplete Immune Cascades. Proc. R. Soc. B Biol. Sci..

[146] Aronstein K.A., Murray K.D., Saldivar E. (2010). Transcriptional Responses in Honey Bee Larvae Infected with Chalkbrood Fungus. BMC Genom..

[147] Lourenço A.P., Florecki M.M., Simões Z.L.P., Evans J.D. (2018). Silencing of *Apis mellifera* Dorsal Genes Reveals Their Role in Expression of the Antimicrobial Peptide Defensin-1. Insect Mol. Biol..

[148] Kingsolver M.B., Huang Z., Hardy R.W. (2013). Insect Antiviral Innate Immunity: Pathways, Effectors, and Connections. J. Mol. Biol..

[149] Feng M., Fei S., Xia J., Labropoulou V., Swevers L., Sun J. (2020). Antimicrobial Peptides as Potential Antiviral Factors in Insect Antiviral Immune Response. Front. Immunol..

[150] Millanta F., Sagona S., Mazzei M., Forzan M., Poli A., Felicioli A. (2019). Phenoloxidase Activity and Haemolymph Cytology in Honeybees Challenged with a Virus Suspension (Deformed Wings Virus DWV) or Phosphate Buffered Suspension (PBS). Cienc. Rural.

[151] González-Santoyo I., Córdoba-Aguilar A. (2012). Phenoloxidase: A Key Component of the Insect Immune System. Entomol. Exp. Appl..

[152] Laughton A.M., Boots M., Siva-Jothy M.T. (2011). The Ontogeny of Immunity in the Honey Bee, *Apis mellifera* L. Following an Immune Challenge. J. Insect Physiol..

[153] Zufelato M.S., Lourenço A.P., Simões Z.L.P., Jorge J.A., Bitondi M.M.G. (2004). Phenoloxidase Activity in *Apis mellifera* Honey Bee Pupae, and Ecdysteroid-Dependent Expression of the Prophenoloxidase MRNA. Insect Biochem. Mol. Biol..

[154] Elias-Neto M., Nascimento A.L.O., Bonetti A.M., Nascimento F.S., Mateus S., Garófalo C.A., Bitondi M.M.G. (2014). Heterochrony of Cuticular Differentiation in Eusocial Corbiculate Bees. Apidologie.

[155] Washburn J.O., Kirkpatrick B.A., Volkman L.E. (1996). Insect Protection against Viruses. Nature.

[156] Dubovskiy I.M., Kryukova N.A., Glupov V.V., Ratcliffe N.A. (2016). Encapsulation and Nodulation in Insects. Invertebr. Surviv. J..

[157] Schmid M.R., Brockmann A., Pirk C.W.W., Stanley D.W., Tautz J. (2008). Adult Honeybees (*Apis mellifera* L.) Abandon Hemocytic, but Not Phenoloxidase-Based Immunity. J. Insect Physiol..

[158] Hystad E.M., Salmela H., Amdam G.V., Münch D. (2017). Hemocyte-Mediated Phagocytosis Differs between Honey Bee (*Apis mellifera*) Worker Castes. PLoS ONE.

[159] Wilson-Rich N., Dres S.T., Starks P.T. (2008). The Ontogeny of Immunity: Development of Innate Immune Strength in the Honey Bee (*Apis mellifera*). J. Insect Physiol..

[160] Schmid-Hempel P. (1995). Parasites and Their Social Hosts. Apidologie.

[161] Desai S.D., Currie R.W. (2015). Genetic Diversity within Honey Bee Colonies Affects Pathogen Load and Relative Virus Levels in Honey Bees, *Apis mellifera* L.. Behav. Ecol. Sociobiol..

[162] Evans J.D., Spivak M. (2010). Socialized Medicine: Individual and Communal Disease Barriers in Honey Bees. J. Invertebr. Pathol..

[163] Castella G., Chapuisat M., Moret Y., Christe P. (2008). The Presence of Conifer Resin Decreases the Use of the Immune System in Wood Ants. Ecol. Entomol..

[164] Wilson-Rich N., Spivak M., Fefferman N.H., Starks P.T. (2009). Genetic, Individual, and Group Facilitation of Disease Resistance in Insect Societies. Annu. Rev. Entomol..

[165] Harpur B.A., Chernyshova A., Soltani A., Tsvetkov N., Mahjoorighasrodashti M., Xu Z., Zayed A. (2014). No Genetic Tradeoffs between Hygienic Behaviour and Individual Innate Immunity in the Honey Bee, *Apis mellifera*. PLoS ONE.

[166] Pérez-Sato J.A., Chline N., Martin S.J., Hughes W.O.H., Ratnieks F.L.W. (2009). Multi-Level Selection for Hygienic Behaviour in Honeybees. Heredity.

[167] Oxley P.R., Spivak M., Oldroyd B.P. (2010). Six Quantitative Trait Loci Influence Task Thresholds for Hygienic Behaviour in Honeybees (*Apis mellifera*). Mol. Ecol..

[168] Le Conte Y., Meixner M.D., Brandt A., Carreck N.L., Costa C., Mondet F., Büchler R. (2020). Geographical Distribution and Selection of European Honey Bees Resistant to *Varroa destructor*. Insects.

[169] Pusceddu M., Cini A., Alberti S., Salaris E. (2021). Honey Bees Increase Social Distancing When Facing the Ectoparasite *Varroa destructor*. Sci. Adv..

[170] Conroy T.E., Holman L. (2022). Social Immunity in the Honey Bee: Do Immune-Challenged Workers Enter Enforced or Self-Imposed Exile?. Behav. Ecol. Sociobiol..

[171] Starks P., Blackie C., Seeley T. (2000). Fever in Honeybee Colonies. Naturwissenschaften.

[172] Goblirsch M., Warner J.F., Sommerfeldt B.A., Spivak M. (2020). Social Fever or General Immune Response? Revisiting an Example of Social Immunity in Honey Bees. Insects.

[173] Bordier C., Dechatre H., Suchail S., Peruzzi M., Soubeyrand S., Pioz M., Pélissier M., Crauser D., Le Conte Y., Alaux C. (2017). Colony Adaptive Response to Simulated Heat Waves and Consequences at the Individual Level in Honeybees (*Apis mellifera*). Sci. Rep..

[174] Ilyasov R.A., Gaifullina L.R., Saltykova E.S., Poskryakov A.V., Nikolaenko A.G. (2013). Defensins in the Honeybee Antiinfectious Protection. J. Evol. Biochem. Physiol..

[175] Harwood G., Salmela H., Freitak D., Amdam G. (2021). Social Immunity in Honey Bees: Royal Jelly as a Vehicle in Transferring Bacterial Pathogen Fragments between Nestmates. J. Exp. Biol..

[176] Borba R.S., Klyczek K.K., Mogen K.L., Spivak M. (2015). Seasonal Benefits of a Natural Propolis Envelope to Honey Bee Immunity and Colony Health. J. Exp. Biol..

[177] Drescher N., Klein A.M., Neumann P., Yañez O., Leonhardt S.D. (2017). Inside Honeybee Hives: Impact of Natural Propolis on the Ectoparasitic Mite *Varroa destructor* and Viruses. Insects.

[178] Simone-Finstrom M.D., Spivak M. (2012). Increased Resin Collection after Parasite Challenge: A Case of Self-Medication in Honey Bees?. PLoS ONE.

[179] Ripari N., Sartori A.A., Honorio M.D.S., Conte F.L., Tasca K.I., Santiago K.B., Sforcin J.M. (2021). Propolis Antiviral and Immunomodulatory Activity: A Review and Perspectives for COVID-19 Treatment. J. Pharm. Pharmacol..

[180] Contreras-Garduño J., Lanz-Mendoza H., Franco B., Nava A., Pedraza-Reyes M., Canales-Lazcano J. (2016). Insect Immune Priming: Ecology and Experimental Evidences. Ecol. Entomol..

[181] Harwood G., Amdam G., Freitak D. (2019). The Role of Vitellogenin in the Transfer of Immune Elicitors from Gut to Hypopharyngeal Glands in Honey Bees (*Apis mellifera*). J. Insect Physiol..

[182] Sadd B.M., Kleinlogel Y., Schmid-Hempel R., Schmid-Hempel P. (2005). Trans-Generational Immune Priming in a Social Insect. Biol. Lett..

[183] DeGrandi-Hoffman G., Chen Y., Huang E., Huang M.H. (2010). The Effect of Diet on Protein Concentration, Hypopharyngeal Gland Development and Virus Load in Worker Honey Bees (*Apis mellifera* L.). J. Insect Physiol..

[184] Dolezal T., Krejcova G., Bajgar A., Nedbalova P., Strasser P. (2019). Molecular Regulations of Metabolism during Immune Response in Insects. Insect Biochem. Mol. Biol..

[185] Walton A., Toth A.L., Dolezal A.G. (2021). Developmental Environment Shapes Honeybee Worker Response to Virus Infection. Sci. Rep..

[186] Di Pasquale G., Salignon M., Le Conte Y., Belzunces L.P., Decourtye A., Kretzschmar A., Suchail S., Brunet J.-L., Alaux C. (2013). Influence of Pollen Nutrition on Honey Bee Health: Do Pollen Quality and Diversity Matter?. PLoS ONE.

[187] Branchiccela B., Castelli L., Corona M., Díaz-Cetti S., Invernizzi C., Martínez de la Escalera G., Mendoza Y., Santos E., Silva C., Zunino P. (2019). Impact of Nutritional Stress on the Honeybee Colony Health. Sci. Rep..

[188] Leonard S.P., Powell J.E., Perutka J., Geng P., Heckmann L.C., Horak R.D., Davies B.W., Ellington A.D., Barrick J.E., Moran N.A. (2020). Engineered Symbionts Activate Honey Bee Immunity and Limit Pathogens. Science.

[189] Kuster R.D., Boncristiani H.F., Rueppell O. (2014). Immunogene and Viral Transcript Dynamics during Parasitic *Varroa destructor* Mite Infection of Developing Honey Bee (*Apis mellifera*) Pupae. J. Exp. Biol..

[190] Navajas M., Migeon A., Alaux C., Martin-Magniette M.L., Robinson G.E., Evans J.D., Cros-Arteil S., Crauser D., Le Conte Y. (2008). Differential Gene Expression of the Honey Bee *Apis mellifera* Associated with *Varroa destructor* Infection. BMC Genom..

[191] Castelli L., García M.L.G., Dalmon A., Arredondo D., Antúnez K., Invernizzi C., Reynaldi F.J., Le Conte Y., Beaurepaire A. (2021). Intra-Colonial Viral Infections in Western Honey Bees (*Apis mellifera*). Microorganisms.

[192] DaPalma T., Doonan B.P., Trager N.M., Kasman L.M. (2010). A Systematic Approach to Virus-Virus Interactions. Virus Res..

[193] Maori E., Lavi S., Mozes-Koch R., Gantman Y., Peretz Y., Edelbaum O., Tanne E., Sela I. (2007). Isolation and Characterization of Israeli Acute Paralysis Virus, a Dicistrovirus Affecting Honeybees in Israel: Evidence for Diversity Due to Intra- and Inter-Species Recombination. J. Gen. Virol..

[194] Mordecai G.J., Brettell L.E., Martin S.J., Dixon D., Jones I.M., Schroeder D.C. (2016). Superinfection Exclusion and the Long-Term Survival of Honey Bees in Varroa-Infested Colonies. ISME J..

[195] Gusachenko O.N., Woodford L., Balbirnie-Cumming K., Evans D.J. (2021). First Come, First Served: Superinfection Exclusion in Deformed Wing Virus Is Dependent upon Sequence Identity and Not the Order of Virus Acquisition. ISME J..

[196] Nickbakhsh S., Mair C., Matthews L., Reeve R., Johnson P.C.D., Thorburn F., Von Wissmann B., Reynolds A., McMenamin J., Gunson R.N. (2019). Virus-Virus Interactions Impact the Population Dynamics of Influenza and the Common Cold. Proc. Natl. Acad. Sci. USA.

[197] Biancotto A., Iglehart S.J., Lisco A., Vanpouille C., Grivel J.C., Lurain N.S., Reichelderfer P.S., Margolis L.B. (2008). Upregulation of Human Cytomegalovirus by HIV Type 1 in Human Lymphoid Tissue Ex Vivo. AIDS Res. Hum. Retrovir..

[198] Cox-Foster D.L., Conlan S., Holmes E.C., Palacios G., Evans J.D., Moran N.A., Quan P., Briese T., Hornig M., Geiser D.M. (2007). A Metagenomic Survey of Microbes in Honey Bee Colony Collapse Disorder. Science.

[199] Dainat B., Evans J.D., Chen Y.P., Gauthier L., Neumann P. (2012). Predictive Markers of Honey Bee Colony Collapse. PLoS ONE.

[200] Mordecai G.J., Wilfert L., Martin S.J., Jones I.M., Schroeder D.C. (2016). Diversity in a honey bee pathogen: First report of a third master variant of the Deformed Wing Virus quasispecies. ISME J..

[201] De Miranda J.R., Brettell L.E., Chejanovsky N., Childers A.K., Dalmon A., Deboutte W., de Graaf D.C., Doublet V., Gebremedhn H., Genersch E. (2022). Cold case: The disappearance of Egypt bee virus, a fourth distinct master strain of deformed wing virus linked to honeybee mortality in 1970’s Egypt. Virol. J..

[202] Norton A.M., Remnant E.J., Buchmann G., Beekman M. (2020). Accumulation and Competition Amongst Deformed Wing Virus Genotypes in Naïve Australian Honeybees Provides Insight into the Increasing Global Prevalence of Genotype B. Front. Microbiol..

[203] McMahon D.P., Natsopoulou M.E., Doublet V., Fürst M., Weging S., Brown M.J.F., Gogol-Döring A., Paxton R.J. (2016). Elevated Virulence of an Emerging Viral Genotype as a Driver of Honeybee Loss. Proc. R. Soc. B Biol. Sci..

[204] Natsopoulou M.E., McMahon D.P., Doublet V., Frey E., Rosenkranz P., Paxton R.J. (2017). The Virulent, Emerging Genotype B of Deformed Wing Virus Is Closely Linked to Overwinter Honeybee Worker Loss. Sci. Rep..

[205] Al Naggar Y., Paxton R.J. (2021). The novel insecticides flupyradifurone and sulfoxaflor do not act synergistically with viral pathogens in reducing honey bee (*Apis mellifera*) survival but sulfoxaflor modulates host immunocompetence. Microb. Biotechnol..

[206] Tehel A., Vu Q., Bigot D., Gogol-Döring A., Koch P., Jenkins C., Doublet V., Theodorou P., Paxton R. (2019). The Two Prevalent Genotypes of an Emerging Infectious Disease, Deformed Wing Virus, Cause Equally Low Pupal Mortality and Equally High Wing Deformities in Host Honey Bees. Viruses.

[207] Zioni N., Soroker V., Chejanovsky N. (2011). Replication of *Varroa destructor* Virus 1 (VDV-1) and a *Varroa destructor* Virus 1-Deformed Wing Virus Recombinant (VDV-1-DWV) in the Head of the Honey Bee. Virology.

[208] Ryabov E.V., Childers A.K., Chen Y., Madella S., Nessa A., VanEngelsdorp D., Evans J.D. (2017). Recent Spread of *Varroa destructor* Virus-1, a Honey Bee Pathogen, in the United States. Sci. Rep..

[209] Woodford L., Evans D.J. (2021). Deformed Wing Virus: Using Reverse Genetics to Tackle Unanswered Questions about the Most Important Viral Pathogen of Honey Bees. FEMS Microbiol. Rev..

[210] Amiri E., Herman J.J., Strand M.K., Tarpy D.R., Rueppell O. (2020). Egg Transcriptome Profile Responds to Maternal Virus Infection in Honey Bees, *Apis mellifera*. Infect. Genet. Evol..

[211] Dubois E., Dardouri M., Schurr F., Cougoule N., Sircoulomb F., Thiéry R. (2020). Outcomes of Honeybee Pupae Inoculated with Deformed Wing Virus Genotypes A and B. Apidologie.

[212] Mondet F., de Miranda J.R., Kretzschmar A., Le Conte Y., Mercer A.R. (2014). On the Front Line: Quantitative Virus Dynamics in Honeybee (*Apis mellifera* L.) Colonies along a New Expansion Front of the Parasite *Varroa destructor*. PLoS Pathog..

[213] BAILEY L., BALL B.V., PERRY J.N. (1981). The Prevalence of Viruses of Honey Bees in Britain. Ann. Appl. Biol..

[214] Carrillo-Tripp J., Dolezal A.G., Goblirsch M.J., Miller W.A., Toth A.L., Bonning B.C. (2016). In Vivo and in Vitro Infection Dynamics of Honey Bee Viruses. Sci. Rep..

[215] Nayak A., Berry B., Tassetto M., Kunitomi M., Acevedo A., Deng C., Krutchinsky A., Gross J., Antoniewski C., Andino R. (2010). Cricket Paralysis Virus Antagonizes Argonaute 2 to Modulate Antiviral Defense in Drosophila. Nat. Struct. Mol. Biol..

[216] Bailey L., Milne R.G. (1969). The Multiplication Regions and Interaction of Acute and Chronic Bee-Paralysis Viruses in Adult Honey Bees. J. Gen. Virol..

[217] Olivier V., Massou I., Celle O., Blanchard P., Schurr F., Ribière M., Gauthier M. (2008). In Situ Hybridization Assays for Localization of the Chronic Bee Paralysis Virus in the Honey Bee (*Apis mellifera*) Brain. J. Virol. Methods.

[218] Deng Y., Yang S., Zhao H., Diao Q., Hou C. (2021). IAPV-Induced Paralytic Symptoms Associated with Tachypnea via Impaired Tracheal System Function. Int. J. Mol. Sci..

[219] Konig C., Schmid-Hempel P. (1995). Foraging Activity and Immunocompetence in Workers of the Bumble Bee, *Bombus terrestris* L.. Proc. R. Soc. B Biol. Sci..

[220] Moret Y., Schmid-Hempel P. (2000). Survival for Immunity: The Price of Immune System Activation for Bumblebee Workers. Science.

[221] Mobley M.W., Gegear R.J. (2018). Immune-Cognitive System Connectivity Reduces Bumblebee Foraging Success in Complex Multisensory Floral Environments. Sci. Rep..

[222] Riessberger-Gallé U., Hernández López J., Schuehly W., Crockett S., Krainer S., Crailsheim K. (2015). Immune Responses of Honeybees and Their Fitness Costs as Compared to Bumblebees. Apidologie.

[223] Vilcinskas A. (2013). Evolutionary Plasticity of Insect Immunity. J. Insect Physiol..

[224] Al Naggar Y., Paxton R.J. (2020). Mode of transmission determines the virulence of black queen cell virus in adult honey bees, posing a future threat to bees and apiculture. Viruses.

[225] Le Clec’h W., Dittmer J., Raimond M., Bouchon D., Sicard M. (2017). Phenotypic shift in Wolbachia virulence towards its native host across serial horizontal passages. Proc. R. Soc. B Biol. Sci..

[226] Lark K.K., Un Y.C., Hwan S.C., Jung S.L., Bin Lee W., Kim J., Jeong K., Shim J., Kim-Ha J., Kim Y.J. (2007). Down-Regulation of NF-ΚB Target Genes by the AP-1 and STAT Complex during the Innate Immune Response in Drosophila. PLoS Biol..

[227] De Smet L., Ravoet J., Wenseleers T., de Graaf D.C. (2017). Expression of Key Components of the RNAi Machinery Are Suppressed in *Apis mellifera* That Suffer a High Virus Infection. Entomol. Sci..

[228] Adamo S.A. (2017). Stress Responses Sculpt the Insect Immune System, Optimizing Defense in an Ever-Changing World. Dev. Comp. Immunol..

[229] Adamo S.A. (2017). The Stress Response and Immune System Share, Borrow, and Reconfigure Their Physiological Network Elements: Evidence from the Insects. Horm. Behav..

[230] Ravoet J., De Smet L., Wenseleers T., de Graaf D.C. (2015). Vertical Transmission of Honey Bee Viruses in a Belgian Queen Breeding Program. BMC Vet. Res..

[231] Shen M., Cui L., Ostiguy N., Cox-Foster D. (2005). Intricate Transmission Routes and Interactions between Picorna-like Viruses (Kashmir Bee Virus and Sacbrood Virus) with the Honeybee Host and the Parasitic Varroa Mite. J. Gen. Virol..

[232] Liu X., Zhang Y., Yan X., Han R. (2010). Prevention of Chinese Sacbrood Virus Infection in *Apis cerana* Using RNA Interference. Curr. Microbiol..

[233] Ryabov E.V., Christmon K., Heerman M.C., Posada-Florez F., Harrison R.L., Chen Y., Evans J.D. (2020). Development of a Honey Bee RNA Virus Vector Based on the Genome of a Deformed Wing Virus. Viruses.

[234] Jin L., Mehmood S., Zhang G., Song Y., Su S., Huang S., Huang H., Zhang Y., Geng H., Huang W.F. (2020). Visualizing Sacbrood Virus of Honey Bees via Transformation and Coupling with Enhanced Green Fluorescent Protein. Viruses.

